# Electroacupuncture Preconditioning Ameliorates the Ischemic Microenvironment to Improve Long-Term Potentiation in Chronic Cerebral Hypoperfusion Rats With MGE Neural Progenitor Transplantation

**DOI:** 10.1155/np/9933756

**Published:** 2025-09-10

**Authors:** Danping Li, Juan Li, Luting Chen, Qiongfang Wu, Min Lu, Xiaohua Han, Hong Chen

**Affiliations:** ^1^Department of Rehabilitation Medicine, Tongji Hospital, Tongji Medical College, Huazhong University of Science and Technology, Wuhan, China; ^2^Department of Rehabilitation Medicine, Zhongda Hospital, Southeast University, Nanjing, China; ^3^Department of Rehabilitation Medicine, General Hospital of the Yangtze River Shipping, Wuhan, China; ^4^Hubei Key Laboratory of Neural Injury and Functional Reconstruction, Huazhong University of Science and Technology, Wuhan, China

**Keywords:** cell transplantation, chronic cerebral hypoperfusion, electroacupuncture, long-term potentiation, MGE neural progenitor, vascular cognitive impairment

## Abstract

**Background:** Vascular cognitive impairment (VCI) is the second most common type of cognitive impairment in the world after Alzheimer's disease (AD). At present, there is no specific drug for VCI. This study aims to confirm the role of electroacupuncture (EA) preconditioning in improving the long-term potentiation (LTP) of chronic cerebral hypoperfusion (CCH) rats with human embryonic stem cell (hESC)-derived medial ganglionic eminence (MGE) neural progenitor transplantation and to investigate its possible mechanism.

**Methods:** Rats with two-vessel occlusion (2VO) were selected as models for the study of VCI. The rats in the 2VO + cell + EA group were given EA for 7 days after modeling. On the 7^th^ day, MGE neural progenitors were transplanted into the hippocampus of CCH rats. 2 weeks after transplantation, we detected the expressions of Iba1, CX3CL1/CX3CR1, Bcl2/Bax, brain-derived neurotrophic factor (BDNF), and tyrosine receptor kinase B (TrkB) in the hippocampus of rats by western blot. Immunofluorescence staining was used to observe the morphologies of microglia and the survival and differentiation of transplanted cells. Microglial morphologies were quantitatively analyzed using the AnalyzeSkeleton. 8 weeks after transplantation, the LTP in the hippocampus of brain slices was detected to evaluate the learning and memory function of the rats with CCH.

**Results:** 2 weeks after transplantation, we observed that MGE neural progenitors survived and differentiated into neurons in the hippocampus of CCH rats. Inflammation and apoptosis appeared in the hippocampus of rats after the interruption of cerebral blood flow. EA preconditioning notably alleviated the inflammatory response and inhibited cell apoptosis in the hippocampus. Moreover, we detected that the expressions of BDNF and TrkB were increased in the hippocampus of rats in the 2VO + cell group and 2VO + cell + EA groups, especially in the 2VO + cell + EA groups. 8 weeks after transplantation, the electrophysiological experiment results showed that the LTP value in the 2VO group was 103.1% ± 2.316%. Compared with the 2VO group, LTP value increased in the 2VO + cell group and 2VO + cell + EA group, which were 136.2% ± 1.603% and 170.8% ± 15.82%, respectively. The increase of LTP value in the 2VO + cell + EA group was more obvious.

**Conclusion:** MGE neural progenitor transplantation improves the LTP of CCH rats, and EA preconditioning can enhance the efficacy of cell transplantation. This enhancement mechanism may be attributed to the effect of EA preconditioning on ameliorating the ischemic microenvironment.

## 1. Introduction

Vascular cognitive impairment (VCI) is a severe syndrome of cognitive dysfunction associated with ischemic and hypoxic damage caused by cerebral vascular disease [1, 2]. VCI accounts for at least 20%–40% of all dementia patients [3]. It is the second most prevalent form of cognitive impairment globally, following Alzheimer's disease (AD) [4]. Patients with VCI commonly present with deficits in learning and memory, as well as psychiatric symptoms like anxiety and depression, along with executive function disorders. In severe cases, they lose the ability to take care of themselves. Therefore, VCI is a serious challenge for global public health. Chronic cerebral hypoperfusion (CCH) is identified as the primary etiological factor of VCI, as long-term hypoperfusion can activate various pathological mechanisms such as neuroinflammation [5], apoptosis [6], and oxidative stress [7], leading to extensive neuronal damage and ultimately contributing to the development and worsening of VCI [2]. Unlike AD, VCI lacks treatment methods approved by the Food and Drug Administration. Currently, the therapeutic approaches and efficacy of VCI are still subject to certain limitations [8–11]. Clinically, drugs targeting AD are often used for the treatment of VCI, such as memantine and cholinesterase inhibitors [11]. However, memantine and cholinesterase inhibitors can only temporarily relieve symptoms and cannot change the course of the disease or reverse nerve damage [12].

The investigation of cell transplantation therapy in VCI has garnered significant attention in contemporary research. Various cell types have been explored, such as induced pluripotent stem cells (iPSCs), embryonic stem cells (ESCs), neural stem cells, and mesenchymal stem cells. The potential mechanisms involved in cell therapy encompass the replacement of damaged neurons, secretion of trophic factors, facilitation of angiogenesis, and reconstruction of neural circuits [13]. Research has demonstrated that stem cell transplantation has the potential to ameliorate cognitive impairment following cerebral ischemia [14, 15]. Medial ganglionic eminence (MGE) is a ventral terminal brain structure located in the middle stage of embryonic development. MGE cells can generate gamma-aminobutyric acid (GABA)ergic interneurons that migrate to the striata and cerebral cortex, GABAergic projection neurons that migrate to the globus pallidus, and cholinergic neurons remaining in the basal end of the brain [16]. MGE cells have potential value in treating neurological diseases [17, 18]. After transplanting into the host, MGE cells can differentiate into neurons and migrate widely, integrating into the neural circuit [19]. The advantages of MGE cells as transplanted cells include: (1) In the adult brain, the migration ability of some neural stem cells is limited [20], while MGE cells can migrate widely after transplantation [21]. Our research group has also observed this phenomenon of cell migration in previous experiments [22]; (2) MGE cell transplantation is relatively safe, and teratoma formation has not been observed in the related studies of MGE cells [21, 23]; (3) Even in harsh environments, MGE cells can still survive when transplanted into the host [24]. Most of the MGE cells used in the current study were derived from fetal mice, and the source of transplanted cells will be a big challenge if we want to translate it into clinical practice. Therefore, human ESCs (hESCs)-derived MGE neural progenitors were selected as the transplanted cells in our study. In addition to the types of transplanted cells, the routes of cell transplantation are also of concern. Cell transplantation routes include direct intracranial injection [25], endovascular injection (intra-arterial injection and intravenous injection) [26], and intranasal injection [27]. Studies have shown that directly injecting stem cells into specific areas of the brain through stereotactic technology seems to be the most effective treatment route [26, 28]. Stereotactic injection of cells into the brain can directly target damaged areas and reduce cell loss during long-term migration [29]. However, the problem with cell transplantation is that the adverse cerebral ischemic microenvironment, such as inflammation, apoptosis, and oxidative stress, hinders the survival of transplanted cells and consequently restricts the efficacy of transplantation. The local microenvironment is an important factor influencing the fate and function of transplanted cells in vivo [30, 31]. Therefore, an intervention that promotes graft survival and improves the efficacy of transplantation is needed.

Electroacupuncture (EA) is a routine rehabilitation therapy for stroke patients. Studies indicated that EA significantly alleviated neurological deficits and improved cognitive function after ischemic stroke [32, 33]. EA could inhibit microglia-mediated neuroinflammation after cerebral ischemia and play a neuroprotective role [34, 35]. In addition, EA could also inhibit the NF-κB signaling pathway to play an antiapoptotic role [36]. Zhao et al. [37] discovered that acupuncture modulated the brain microenvironment to further improve the therapeutic effect of neural stem cells in AD mice. Based on the regulatory impact of EA on the brain microenvironment, our research team aims to employ EA preconditioning to improve the ischemic microenvironment and foster a good living environment for transplanted cells before transplantation. Acupuncture at Baihui (GV20) and Dazhui (GV14) could significantly improve the cognitive function of VCI rats by regulating cerebral blood flow, inhibiting inflammation, and inhibiting apoptosis [38–40], so we chose them as treatment points in this study.

Long-term potentiation (LTP) is a phenomenon of continuous enhancement of synaptic strength in the hippocampus and is regarded as the cellular basis of learning and memory [41, 42]. It has been found that CCH can lead to impaired LTP [43]. Brain-derived neurotrophic factor (BDNF) is an important factor for the development, growth, and maintenance of neurons in the nervous system, and is involved in synaptic plasticity, learning, and memory [44]. BDNF and its receptor tyrosine receptor kinase B (TrkB) are important regulators of synaptic transmission and LTP in the hippocampus and other brain regions [45]. Activation of the BDNF/TrkB signaling pathway rescued impaired LTP and reversed synaptic damage and memory impairment [46]. Studies have shown that EA can promote the synaptic transmission efficiency of rats after cerebral ischemia, alleviate LTP injury, and improve learning and memory impairment [47].

Based on the above discussion, this study aims to examine how EA ameliorates the ischemic microenvironment by modulating the inflammatory response and inhibiting apoptosis. Additionally, we evaluated the expression of BDNF and TrkB in the hippocampus of rats and whether EA preconditioning can enhance the efficacy of cell transplantation and improve the LTP of CCH rats.

## 2. Materials and Methods

### 2.1. hESCs Culture and Generation of MGE Neural Progenitors

The cells used in our study were hESCs lines (hM3Dq-KORD, P30−45, WiCell Research Institute) expressing mCherry fluorescent protein. hESCs were cultured with mouse embryonic fibroblasts (MEFs) as a feeder layer in a 6-well plate and passaged weekly as mentioned above [22, 48, 49]. hESCs medium contained 78 mL DMEM/F12(Gibco, C11330500BT), 20 mL knockout serum replacer (Gibco, 10828028), 1 mL MEM NEAA (100x) (Gibco, 11140050), 1 mL Glutmax-I supplement (Gibco, 35050061), 0.7 μL β-mercaptoethanol (Sigma–Aldrich, M3148), and 8 ng/mL basic fibroblast growth factor (Peprotech, 100–18 B).

By a dual SMAD inhibition protocol, hESCs were induced to differentiate into MGE neural progenitors [48, 49]. Neural differentiation medium included 49 mL DMEM/F-12 (Gibco, 11330–032), 49 mL neurobasal medium (1x) (Gibco, 21103049), 1 mL N2 supplement (100x) (Gibco, 17502048), and 1 mL MEM NEAA (100x) (Gibco, 11140050). The experimental procedure consisted of four distinct stages: the first stage (Day 1–7) was to induce hESCs to differentiate into neuroepithelial cells. During this stage, 2 µM DMH1 (Selleck, S7146), 2 µM XAV939 (Selleck, S1180), and 2 µM SB431542 (Selleck, S1067) were added to the medium to inhibit the relevant signal pathways. In the second stage (Day 8–14), MGE neural progenitors were differentiated from neuroepithelial cells. In addition to the above three small molecules, we supplemented the culture medium with 0.5 µM SAG (Selleck, S6384), a sonic hedgehog (SHH) signal pathway agonist. In the third stage (Day 14–20), MGE neural progenitors in the form of neurospheres were suspended in T25 bottles for culture. 2 mL B27 (Gibco, 12587010) and 0.5 µM SAG were added to the differentiation medium during this period. In the fourth stage (Day 21–35), the accutase (Innovative Cell Technologies, AT104)-dissociated neurospheres were inoculated on poly-l-ornithine/laminin-coated coverslips on day 21 to observe the differentiation of MGE neural progenitors into neurons in vitro. At this stage, 10 ng/mL IGF-1 (Peprotech, 100–11), 10 ng/mL GDNF (Peprotech, 450–10), 10 ng/mL BDNF (Peprotech, 450–02), 1 μM cAMP (Sigma–Aldrich, D-0260), 0.5 μM SAG, and B27 were added to the differentiation medium (Figure S1A). The cellular morphologies were observed under the microscope every day.

### 2.2. Animals and Experimental Group

Male Sprague–Dawley (SD) rats (6–8 weeks old, 280–310 g) were raised in the Experimental Animal Center of Tongji Hospital, Tongji Medical College, Huazhong University of Science and Technology. Every three rats were placed in a cage at 25 ± 2°C room temperature and kept in a 12 h automatic light/dark cycle with free intake of water and food. There were five different groups of rats divided randomly: sham operation (Sham) group, two-vessel occlusion model (2VO) group, 2VO model with medium transplantation (2VO + medium) group, 2VO model with cell transplantation (2VO + cell) group, and 2VO model with cell transplantation with EA preconditioning (2VO + cell + EA) group. The experiment was conducted with the approval of the Experimental Animal Ethics Committee of Tongji Hospital, Tongji Medical College, Huazhong University of Science and Technology (TJH-202111014). For each experimental endpoint (western blot, immunofluorescence staining, and electrophysiological recording), animals were randomly assigned to separate cohorts. No animals were used for multiple assays, in order to avoid pseudoreplication and to ensure biological independence across datasets.

### 2.3. Establishment of the 2VO Model

The 2VO model was established based on the method described in the previous study [50]. After the rats were anesthetized, the neck hair was shaved off. Place the rats in their supine position and fix them on the operation table. Disinfect the skin on the neck three times and make a cut of about 2 cm along the middle of the neck. The subcutaneous fascia and superficial muscle groups were bluntly isolated, and the bilateral common carotid arteries were exposed. Carefully separate the common carotid artery with ophthalmic tweezers to prevent excessive stimulation of the vagus nerve. The proximal and distal ends of the common carotid artery were ligated with 4–0 sterile medical sutures and subsequently cut at the midpoint of the two ligation threads to interrupt cerebral blood flow permanently. After suturing the skin, disinfect the wound with iodophor twice. Throughout the entire surgical process, the anal temperature of the rats was maintained at 37 ± 0.2°C. Only the common carotid artery was isolated without ligation in the Sham group.

### 2.4. EA Preconditioning

Rats in the 2VO + cell + EA group were treated with EA for 7 days on the first day after modeling. Select GV20 and GV14 as acupuncture points, as described previously [50] (Figure S2). GV20 is located in the middle of the parietal bone, and GV14 is located below the spinous process of the seventh cervical vertebra, respectively, connected to the negative and positive poles of the low-frequency electronic pulse therapy device. The continuous stimulation current was set at 1–2 mA, with a frequency of 2 Hz, and the treatment time was 20 min per day.

### 2.5. Cell Transplantation

On the 7^th^ day of EA, MGE neural progenitors were transplanted into the hippocampus of rats (Figure S3). MGE neural progenitors were dissociated with accutase the day before transplantation to obtain a single-cell suspension (cell concentration: 1 × 10^5^ cells/μL, cell viability >80%). Rats were positioned in the stereoscope after anesthesia. The skull plane of the rats was kept horizontal. With the bregma as the origin, the positions of the hippocampus were marked (A-P: −4 mm, lateral: ±3 mm, D-V: −3 mm) under the guidance of a stereoscope [22]. Then, drill holes in the marked locations. 2.5 μL of cell suspension was injected into each side of the hippocampal area. The whole injection process lasted 5 min. It was necessary to wait 5 min after the injection to prevent cell backflow. Cyclosporine (10 mg/kg/day) was injected subcutaneously 2 days before cell transplantation until the end of the study. It was only the medium that was transplanted in the 2VO + medium group. The surgeon was blinded to treatment.

### 2.6. Tissue Processing

The rats were perfused successively with 0.9% NaCl solution and precooled 4% paraformaldehyde after being successfully anesthetized (2% pentobarbital sodium, 40 mg/kg, intraperitoneally). Then, we removed the brain tissues from the rats and incubated them in 4% paraformaldehyde at 4°C overnight. Subsequently, they were dehydrated with 20% sucrose followed by 30% sucrose. The dehydrated tissues were embedded in the optimum cutting temperature compound and sliced into 30 μm pieces in a frozen microtome. 24-well plates were used to collect the brain slices for immunofluorescence staining.

### 2.7. Immunofluorescence Staining

Coverslip cultures and brain slices were stained with immunofluorescence according to the previous description [51]. The cells were blocked with a staining blocking solution for half an hour at room temperature. Subsequently, primary antibodies were added and incubated overnight at 4°C. Primary antibodies included Rabbit anti-PAX6 (Biolegend, 901301, 1:1000), Rat anti-HOXB4 (DSHB, II2,1:100), Goat anti-SOX1 (R&D, AF3369,1:1000), Rabbit anti-FOXG1 (Abacm, Ab18259,1:1000), Rabbit anti-NKX2.1 (Abacm, Ab76013,1:500), Rabbit anti-GABA (Sigma, A-2052,1:500), Mouse anti-MAP2 (Sigma, M1406,1:1000), Mouse anti-βІІІ-tubulin (Biolegend, T8660,1:1000), Rabbit anti-VACHT (Sigma, SAB4200560,1:500), Mouse anti-human nuclei (HuNu) (Millipore, MAB1281,1:200), Rabbit anti-Iba1 (Takara, 0191974, 1:500). The cells were sequentially treated with the corresponding secondary antibodies for 45 min away from light and Hoechst for 10 min the next day.

### 2.8. Morphological Analysis of Microglia

Microglial morphologies in the hippocampus of brain slices were observed by laser confocal microscope and images were captured. Three fields of each sample were randomly selected for quantitative analysis under a confocal microscope (Olympus, Japan, 40× objective). According to Young and Morrison's protocol [52], the plug-in AnalyzeSkeleton in ImageJ was used to analyze the morphologies of microglia. The values were averaged to take as one value of each sample. Analysis of microglia morphology was completed by an investigator blinded to the group using ImageJ software.

### 2.9. Western Blot Analysis

Rats were decapitated after deep anesthesia (2% pentobarbital sodium, 40 mg/kg, intraperitoneally), and the hippocampus was quickly isolated on ice. Proteins were extracted from hippocampal tissues by homogenizing them in a lysate buffer containing PMSF. The protein concentration of the samples was measured with a BCA kit. SDS–PAGE fractionated proteins and transferred them to PVDF membranes. Primary antibodies including Rabbit anti-Bax (Affinity, AF0120,1:1000), Rabbit anti-Bcl2 (Affinity, AF6139,1:1000), Mouse anti-β-actin (Affinity, T0022,1:1000), Rabbit anti-GAPDH (Affinity, AF7021,1:1000), Rabbit anti-Iba1 (Affinity, DF6442,1:1000), Rabbit anti-CX3CL1 (Affinity, DF1237, 1:1000), Rabbit anti-CX3CR1 (Affinity, DF7096,1:1000), Rabbit anti-BDNF (Abcam, Ab108319, 1:1000), Rabbit anti-TrkB (Abcam, Ab18987, 1:1000) were used to incubate the membranes, and then treated with the HRP-conjugated goat anti-rabbit, goat anti-mouse secondary antibodies the next day. The band was visualized by the ECL detection kit, and the band intensity was analyzed by the Image-Pro Plus.

### 2.10. Electrophysiological Recording

Anesthesia was administered with pentobarbital sodium, and the rats were perfused with ice-cold dissection buffer saturated with 95% O_2_/5% CO_2_. Ice-cold dissection buffer contained (in mM): 234 sucrose, 11 glucose, 24 NaHCO_3_, 2.5 KCl, 1.25 NaH_2_PO_4_, 0.5 CaCl_2_, and 2 MgSO_4_. Brain tissues were removed and 400 µm-thick hippocampal slices were slowly cut horizontally using a vibratome. Hippocampal slices were recovered at 37°C for 1 h in a chamber filled with oxygenated artificial cerebrospinal fluid (ACSF), which contained (in mM): 10 glucose, 126 NaCl, 26 NaHCO_3_, 2.5 KCl, 1.25 NaH_2_PO_4_, 2 CaCl_2_, and 2 MgCl_2_. 1 h later, patch-clamp recordings could be conducted on brain slices that had been placed at room temperature.

Slices were moved to a recording chamber where carbogen-saturated ACSF was continuously supplied. Electrical stimulation was delivered by placing a concentric circular stimulating electrode on the Schaffer collateral of the hippocampus. Glass microelectrode filled with ACSF was placed in the radiation layer of CA1 field for recording field excitatory postsynaptic potential (fEPSP). fEPSP was evoked with an intensity (duration 0.2 ms) that elicited approximately 50% of the maximum amplitude and a frequency of 0.05 Hz. LTP was induced with a high-frequency stimulation (HFS) procedure, which included two consecutive 1-second trains of 100-Hz stimuli, and the interval between trains for 20 s. The percent increase of the average fEPSP slopes during the last 10 min normalized to the average of the slope in the first 10 min of induction was used as the standardized potentiation. The electrical signals were collected by MultiClamp 700B amplifiers, filtered and sampled by Digidata 1440 A, with a filtering frequency of 1 kHz and a sampling frequency of 20 kHz. Clampex 10.2 was used to record data and Clampfit was used to analyze it.

### 2.11. Statistical Analysis

Data were statistically analyzed using GraphPad Prism 8.0 and presented in the form of mean ± standard error of the mean (SEM). One-way analysis of variance (ANOVA) was used for comparison among multiple groups, and Tukey test was used for pairwise comparison between groups. We drew by GraphPad Prism 8.0, ImageJ, and Adobe Illustrator. *p*  < 0.05 represented that the difference was statistically significant.

## 3. Results

### 3.1. hESCs Were Induced to Differentiate Into MGE Neural Progenitors In Vitro

Through the monolayer adhesion culture method, small molecules such as WNT signal pathway inhibitor XAV939, transforming growth factor-β receptor kinase inhibitor SB431542, bone morphogenetic protein signal pathway inhibitor DMH1, and SHH signal pathway agonist SAG were added at different stages to induce hESCs to differentiate gradually into neuroepithelial cells, MGE neural progenitors, and neurons. By the fourth day of hESCs differentiation, notable changes in cell morphology were observed, characterized by distinct boundaries and the formation of concentric neural tube-like structures. By Day 10, rosettes were present in the majority of cell clones. The suspended bright neurospheres were observed on Day 20. By Day 21, the dissociated neurospheres were cultured adherent to the plate, and the nerve fibers were slowly emitted from the neurospheres after 1 h of adhesion. By Day 35, we observed the synaptic connections between neurons (Figure S1B).

**Figure 1:**
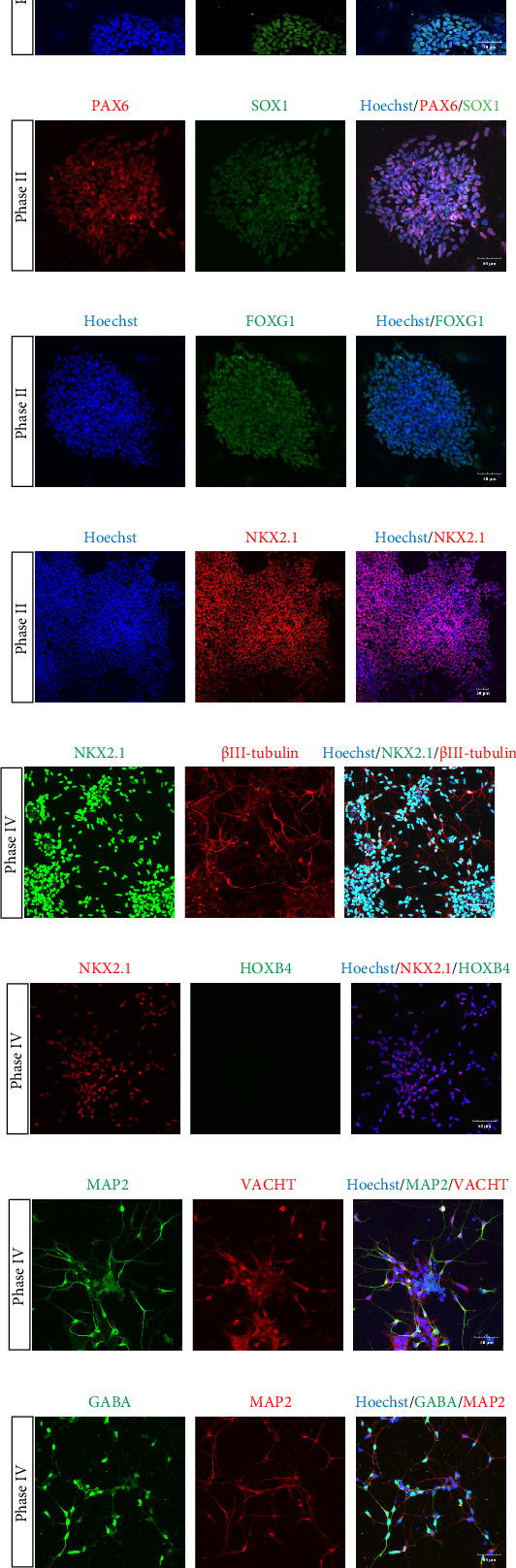
Marker of the process by which human embryonic stem cells differentiate into medial ganglionic eminence (MGE) neural progenitors. (A) The early neuroepithelial cell marker PAX6 was expressed on day 7 of differentiation. (B–D) On the 14^th^ day of differentiation, the neuroepithelial marker SOX1, forebrain marker FOXG1, MGE neural progenitor marker NKX2.1 were detected. (E) Positive expressions of NKX2.1 and early neuron marker βІІІ-tubulin were detected after 2 days of adherent culture. (F) There was no expression of spinal cord marker HOXB4. (G, H) On the 35^th^ day of differentiation, the positive expressions of cholinergic neuron marker VACHT, GABAergic interneuron marker GABA, and mature neuron marker MAP2 were marked (bar = 50 μm).

PAX6, an early neuroepithelial cell marker, was detected on the 7^th^ day of differentiation (Figure 1A), indicating the differentiation of the cells into neuroepithelial cells at this stage. On the 14^th^ day of differentiation, another neuroepithelial cell marker SOX1 (Figure 1B), forebrain marker FOXG1 (Figure 1C), and MGE neural progenitor marker NKX2.1 (Figure 1D) were detected. On the 21^st^ day of differentiation, the dissociated neurospheres were cultured adherently on the plate. The expressions of NKX2.1 and early neuron marker βІІІ-tubulin were identified (Figure 1E), and the expression of spinal cord marker HOXB4 was not observed (Figure 1F) 2 days after adherent culture of the neurospheres. The induction purity of NKX2.1^+^ cells was approximately 89.87 ± 2.46% (Figure S4). On the 35^th^ day, cholinergic neuron marker vesicular acetylcholine transporter (VACHT), GABAergic interneuron marker GABA, and mature neuron marker MAP2 were detected (Figure 1G,H).

### 3.2. The Transplanted MGE Neural Progenitors Survived and Differentiated in the Hippocampus

**Figure 2:**
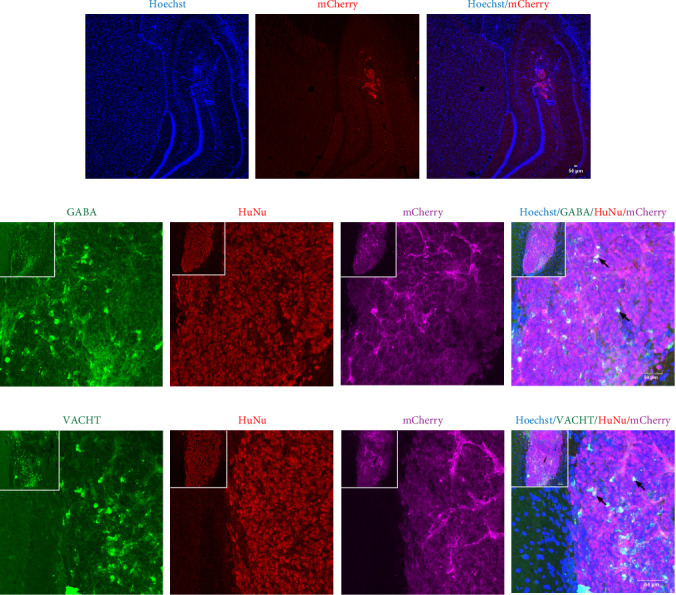
The transplanted cells survived and differentiated into neurons in the hippocampus 2 weeks after transplantation. (A) The mCherry fluorescent proteins were detected in the transplantation area and colocalized with Hoechst. (B, C) Small amounts of GABA and VACHT were expressed in the transplant area, as indicated by black arrows (bar = 50 μm).

MGE neural progenitors were transplanted into the hippocampus of rats, observed for 2 weeks, and identified by immunofluorescence staining. The expression of mCherry fluorescent protein specific to transplanted cells was detected in the hippocampus and colocalized with the cellular nuclear marker Hoechst, as shown in Figure 2A. In addition, we localized the transplanted cells by labeling the human nuclear marker HuNu and mCherry fluorescent protein. We found that the GABAergic interneuron marker GABA and the cholinergic neuron marker VACHT were expressed in a small amount in the transplant area (Figure 2B,C). This suggested that the transplanted cells survived in the hippocampus and several transplanted cells differentiated into neurons.

### 3.3. EA Preconditioning Plays an Anti-Inflammatory Role

**Figure 3:**
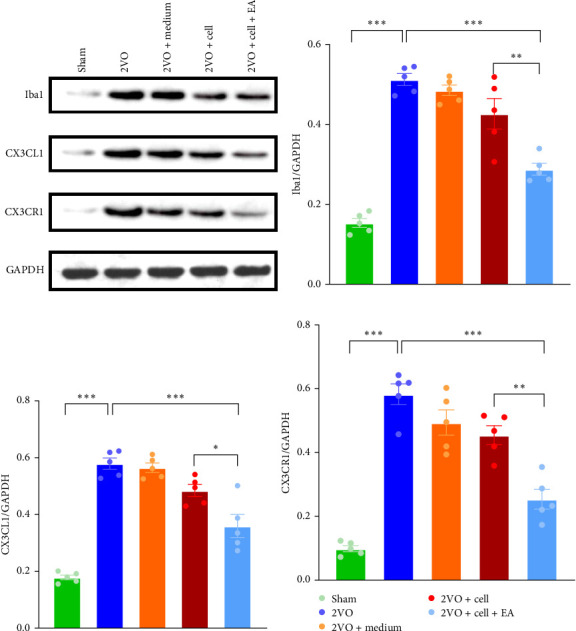
Detect the expressions of microglia marker Iba1, chemokine CX3CL1, CX3CR1 in the hippocampus of each group by western blot. (A) Representative western blot bands. (B–D) Statistical analysis of microglia marker Iba1, chemokine CX3CL1, and its receptor CX3CR1. *⁣*^*∗∗∗*^*p* < 0.001, *⁣*^*∗∗*^*p* < 0.01, *⁣*^*∗*^*p* < 0.05, one-way ANOVA. All data were expressed as mean ± standard error of the mean (SEM; *n* = 5 rats/per group).

2 weeks after transplantation, we detected the expressions of microglial marker Iba1, chemokine CX3CL1, and its receptor CX3CR1 (CX3CL1/CX3CR1) in the hippocampus of rats by western blot (Figure 3A). Iba1 expression was notably higher in the 2VO group than in the Sham group (*p*  < 0.001). Compared with the 2VO group, the expression of Iba1 in the 2VO + cell group was decreased, but the difference was not statistically significant (*p*  > 0.05). Compared with the 2VO group and 2VO + cell group, the expression of Iba1 in the 2VO + cell + EA group was significantly decreased (2VO vs. 2VO + cell + EA: *p*  < 0.001, 2VO + cell vs. 2VO + cell + EA: *p*  < 0.01) (Figure 3B). In the 2VO group, CX3CL1/CX3CR1 expression was higher than in the Sham group (*p*  < 0.001). Compared with the 2VO group, the expressions of CX3CL1 (*p*  < 0.001) and its receptor CX3CR1 (*p*  < 0.001) in the 2VO + cell + EA group were notably decreased, with statistical significance. The expressions of CX3CL1 (*p*  < 0.05) and its receptor CX3CR1 (*p*  < 0.01) in the 2VO + cell + EA group were lower than 2VO + cell group (Figure 3C,D).

**Figure 4:**
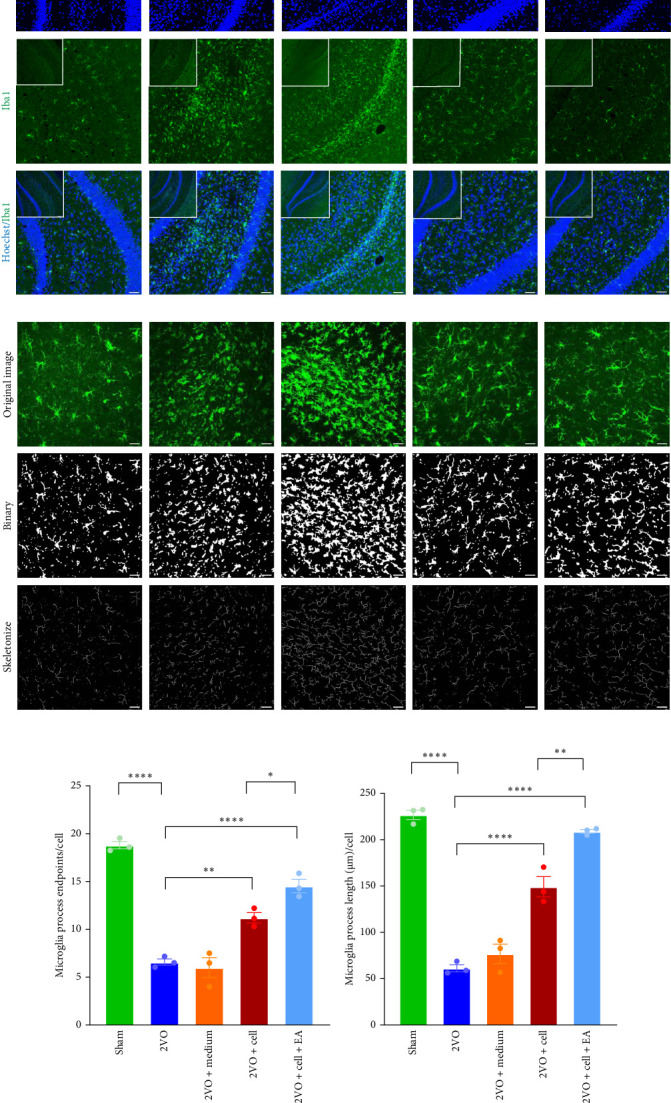
Morphological changes of microglia were assessed by AnalyzeSkeleton. (A) Immunofluorescence images of microglia marker Iba1. (B) ImageJ software was employed to perform AnalyzeSkeleton on microglia; original image, binary image, and skeletonized image from top to bottom. (C) Statistical analysis of microglia process endpoints/cell. (D) Statistical analysis of microglia process length/cell.*⁣*^*∗∗∗∗*^*p* < 0.0001, *⁣*^*∗∗*^*p* < 0.01, *⁣*^*∗*^*p* < 0.05, one-way ANOVA. All data were expressed as mean ± SEM (*n* = 3 rats/per group) (bar = 50 μm).

The microglia marker Iba1 in the hippocampus of rats was labeled by immunofluorescence staining. We found that microglia in the hippocampus of rats were activated after the interruption of cerebral blood flow, and their processes were shortened and reduced, their bodies were rounded, especially in the 2VO and 2VO + medium groups (Figure 4A). ImageJ software was employed to perform AnalyzeSkeleton on microglia in the hippocampus, facilitating the evaluation of morphological changes in microglia (Figure 4B). Compared to the Sham group, microglia process endpoints/cell were decreased (*p*  < 0.0001), and process length/cell was shortened (*p*  < 0.0001) in the 2VO group. Compared to the 2VO group, the microglia process endpoints/cell (2VO vs. 2VO + cell: *p*  < 0.01, 2VO vs. 2VO + cell + EA: *p*  < 0.0001) and process length/cell (2VO vs. 2VO + cell: *p*  < 0.0001, 2VO vs. 2VO + cell + EA: *p*  < 0.0001) were increased in the 2VO + cell and 2VO + cell + EA groups (Figure 4C,D), with the most pronounced changes observed in the 2VO + cell + EA group. These findings indicated that EA preconditioning played a more significant anti-inflammatory effect.

### 3.4. EA Preconditioning Plays an Antiapoptotic Role

**Figure 5:**
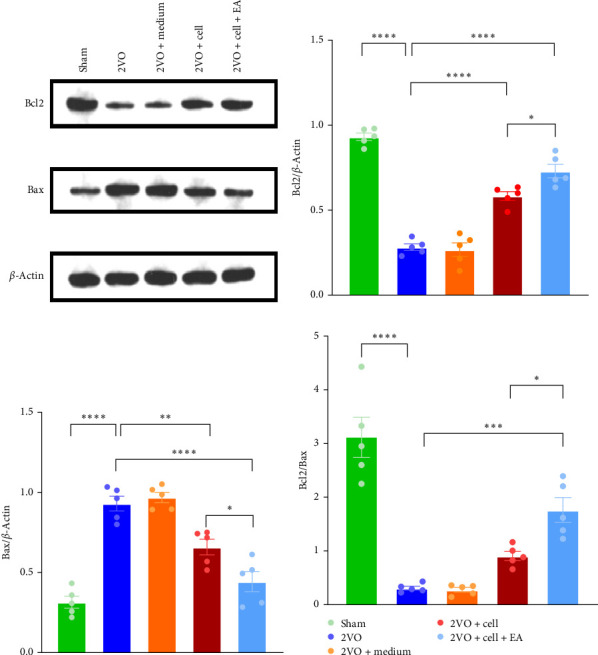
The expressions of antiapoptotic protein Bcl2 and proapoptotic protein Bax were detected by western blot. (A) Representative western blot bands. (B–D) Statistical analysis of the expressions of antiapoptotic protein Bcl2, proapoptotic protein Bax, and the ratio of Bcl2/Bax in each group. *⁣*^*∗∗∗∗*^*p* < 0.0001, *⁣*^*∗∗∗*^*p* < 0.001, *⁣*^*∗∗*^*p* < 0.01, *⁣*^*∗*^*p* < 0.05, one-way ANOVA. All data were expressed as mean ± SEM (*n* = 5 rats/per group).

2 weeks after transplantation, western blot was used to detect the expressions of antiapoptotic protein Bcl2 and proapoptotic protein Bax in the hippocampus of rats (Figure 5A). Compared with the Sham group, the expression of antiapoptotic protein Bcl2 in the 2VO group was downregulated (*p*  < 0.0001). Compared with the 2VO group, the expression of antiapoptotic protein Bcl2 was upregulated in the 2VO + cell group and 2VO + cell + EA group (2VO vs. 2VO + cell: *p*  < 0.0001, 2VO vs. 2VO + cell + EA: *p*  < 0.0001); In the 2VO + cell + EA group, Bcl2 upregulation was more significant than in the 2VO + cell group (*p*  < 0.05) (Figure 5B). The 2VO group showed a significant upregulation in proapoptotic protein Bax relative to the Sham group (*p*  < 0.0001). In the 2VO + cell and 2VO + cell + EA groups, proapoptotic protein Bax levels were lower than in the 2VO group (2VO vs. 2VO + cell: *p*  < 0.01, 2VO vs. 2VO + cell + EA: *p*  < 0.0001); Bax was downregulated in the 2VO + cell + EA group relative to the 2VO + cell group (*p*  < 0.05) (Figure 5C). We also found that the Bcl2/Bax ratio in the 2VO group was decreased than that in the Sham group (*p*  < 0.0001). In contrast to the 2VO group, the Bcl2/Bax ratio was increased in the 2VO + cell (*p*  > 0.05) and 2VO + cell + EA (*p*  < 0.001) groups. Moreover, the 2VO + cell + EA group had a higher Bcl2/Bax ratio than the 2VO + cell group (*p*  < 0.05) (Figure 5D).

### 3.5. EA Preconditioning Combined With MGE Neural Progenitor Transplantation Upregulates the Expression Levels of BDNF and TrkB

**Figure 6:**
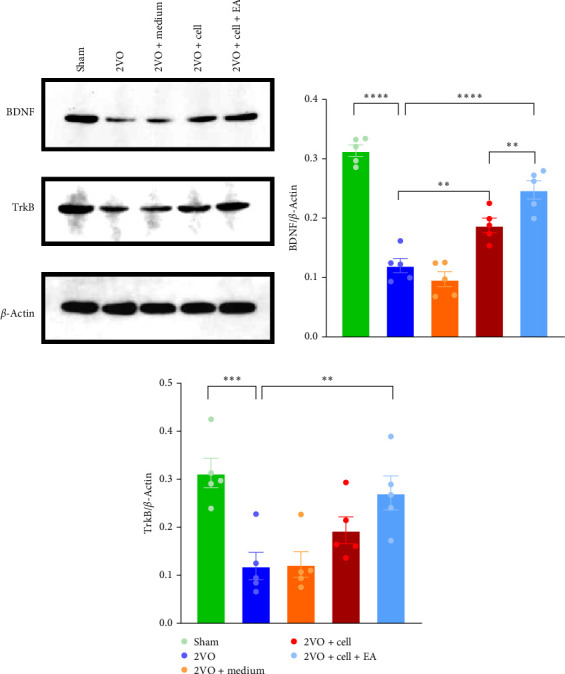
Electroacupuncture (EA) preconditioning combined with MGE neural progenitor transplantation upregulates the expression levels of brain-derived neurotrophic factor (BDNF) and tyrosine receptor kinase B (TrkB). (A) Representative western blot bands. (B–C) Statistical analysis of the expressions of BDNF and TrkB. *⁣*^*∗∗∗∗*^*p*  < 0.0001, *⁣*^*∗∗∗*^*p*  < 0.001, *⁣*^*∗∗*^*p*  < 0.01, one-way ANOVA. All data were expressed as mean ± SEM (*n* = 5 rats/per group).

The expression levels of BDNF and TrkB in the hippocampus of each group were detected by western blot (Figure 6A). Compared to the Sham group, the expressions of BDNF (*p*  < 0.0001) and TrkB (*p*  < 0.001) were downregulated in the 2VO group. The expression of BDNF in the 2VO + cell group (*p*  < 0.01) and 2VO + cell + EA (*p*  < 0.0001) group was upregulated compared with those in the 2VO group, especially in the 2VO + cell + EA group. Meanwhile, we observed a trend of upregulation of TrkB in the 2VO + cell group, but there was no statistical difference between the 2VO group and the 2VO + cell group. However, TrkB expression was significantly increased in the 2VO + cell + EA group compared with the 2VO group (*p*  < 0.01), and the difference was statistically significant (Figure 6B,C).

### 3.6. EA Preconditioning Improves LTP in CCH Rats With MGE Neural Progenitor Transplantation

**Figure 7:**
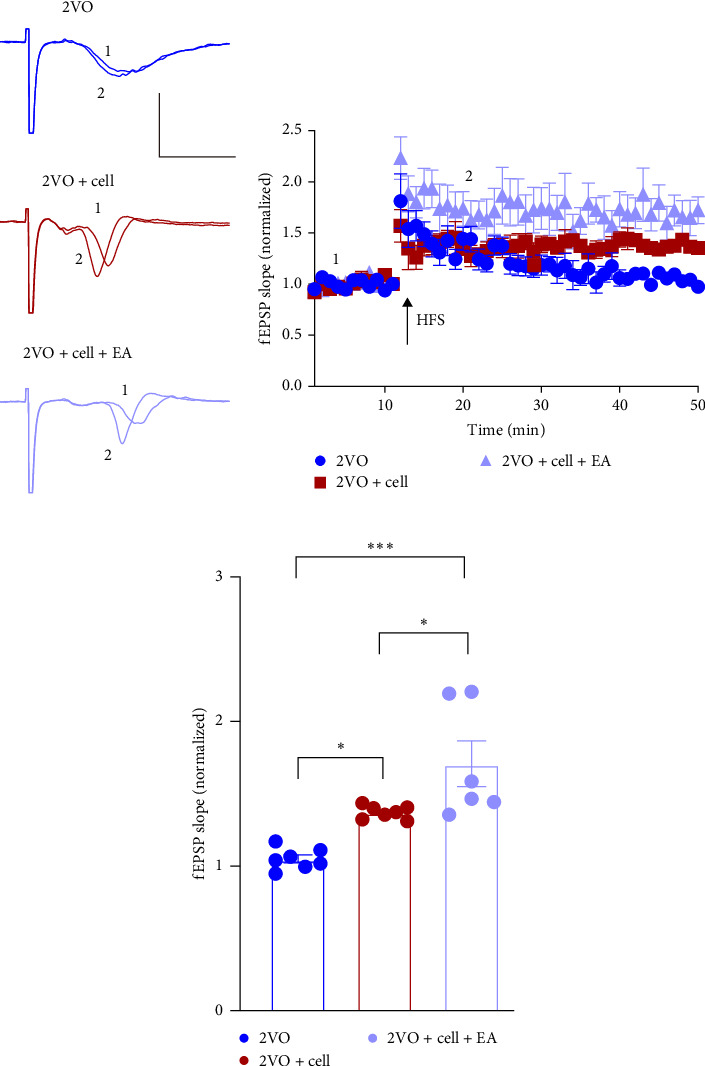
EA preconditioning promoted the improvement of long-term potentiation (LTP) of the CA3-CA1 pathway in the hippocampus of two-vessel occlusion (2VO) rats. (A) Representative traces of the field excitatory postsynaptic potential (fEPSP) during LTP recording. (B) Time-course of fEPSP slopes during LTP recording. (C) The normalized average fEPSP slopes during the last 10 min. 2VO group: *n* = 7 brain slices, 2VO + cell group: *n* = 7 brain slices, 2VO + cell + EA group: *n* = 6 brain slices. The scale bars in (A) were 0.5 mV and 10 ms, respectively. *⁣*^*∗∗∗*^*p* < 0.001, *⁣*^*∗*^*p* < 0.05, One-way ANOVA. All data were expressed as mean ± SEM (*n* = 3 rats/per group).

8 weeks after MGE neural progenitor transplantation, the LTP of the CA3–CA1 pathway in the hippocampus of isolated brain slices was detected by the electrophysiological experiment (Figure 7A,B). This experiment could reflect the learning and memory function of 2VO rats. The normalized fEPSP slope measured in the hippocampus was 103.1% ± 2.316% in the 2VO group. The normalized fEPSP slopes of the 2VO + cell group (136.2% ± 1.603%) and 2VO + cell + EA group (170.8% ± 15.82%) were increased (2VO vs. 2VO + cell: *p*  < 0.05, 2VO vs. 2VO + cell + EA: *p*  < 0.001), especially in the 2VO + cell + EA group. The fEPSP slope in the 2VO + cell + EA group was notably higher than the 2VO + cell group, and the difference was statistically significant (*p*  < 0.05) (Figure 7C).

## 4. Discussion

The 2VO model, a recognized CCH model without cerebral infarction, is known to lead to decreased blood supply in the cortex and hippocampus [53], white matter injury [54], brain capillary lesions [55], and eventually neuron loss. Neurons including the well-known cholinergic neurons and GABAergic interneurons are closely related to cognitive function. Cholinergic neurons are mainly distributed in the basal forebrain. Moreover, they are projectable towards the cortex and hippocampus. Cholinergic signal transduction plays crucial roles in motor learning and spatial memory, such as synaptic plasticity and LTP [56–58]. During development, GABAergic interneurons migrate to the cortex and hippocampus, playing a crucial role in learning and memory processes [59, 60]. Therefore, the regeneration of neurons after brain injury has become a therapeutic target. Nevertheless, the quantity of endogenous neural stem cells is far from sufficient to compensate for the loss of neurons, and these stem cells typically differentiate into glial cells, a population inadequate to compensate for the functional neuron deficit. Therefore, with the rapid development of cell transplantation technology, researchers have tried to transplant exogenous neural stem cells into the brain. Many preclinical studies have suggested that exogenous neural stem cells can supplement or replace injured neurons and save neural function deficits [14, 61–63].

In our study, the selected cells were MGE neural progenitors derived from hESCs. The most worrisome issue with cell transplantation is the potential for tumorigenicity. For instance, ESCs and iPSCs possess the capacity to give rise to malignant tumors or teratomas within the organism due to their totipotent nature. Consequently, there is little research on directly transplanting ESCs or iPSCs, but inducing them into specific cell types [64, 65]. MGE, the pallidus primordium located in the ventral telencephalon of the embryo, is a temporary brain structure that emerges in the mid-phase of embryonic development. It is a vital source of GABAergic interneurons and basal forebrain cholinergic neurons [66]. Studies have shown that MGE cell transplantation improves cognitive function in neurological disorders such as traumatic brain injury [67], AD [18, 24], epilepsy [68], and Fragile X syndrome [17]. Therefore, we selected MGE neural progenitors derived from hESCs for transplantation. In our study, hESCs were induced to differentiate by a dual SMAD inhibition protocol to obtain high-purity NKX2.1^+^ MGE neural progenitors quickly. In addition, our experiment confirmed that MGE neural progenitors could differentiate into cholinergic neurons and GABAergic interneurons in vitro. 2 weeks after transplantation, we detected the coexpression of human nuclei HuNu and mCherry fluorescent protein, which suggested that the cells survived in the transplanted area. Additionally, our investigation found that some transplanted cells expressed GABA, the marker of GABAergic interneurons, and VACHT, the marker of cholinergic neurons. This finding indicated that MGE neural progenitors also differentiated into neurons after transplantation in vivo. Cholinergic neurons and GABAergic interneurons play vital roles in learning and memory, so the emergence of these two types of neurons provides support for the therapeutic value of cell transplantation in cognitive impairment.

Nevertheless, due to the low survival rate of transplanted cells in their host, the transplanted effect of exogenous neural stem cells remains controversial [63, 69]. The reason may be related to the harsh cerebral microenvironment caused by the inflammatory cascade reaction and cell apoptosis after CCH, which is bad for the survival of transplanted cells. EA has been commonly used to treat cerebral ischemia. Therapeutic mechanisms include anti-inflammation, antiapoptosis, promotion of cell proliferation in ischemic areas, and improvement of LTP [70]. Studies have reported that EA treatment for 7 days immediately after cerebral ischemia can improve the ischemic microenvironment and achieve therapeutic effects [71, 72]. Therefore, to create a suitable environment for the transplanted cells, we plan to conduct EA from the first day after molding and continue for 7 days.

Microglia are resident immune cells of the central nervous system that monitor the surrounding environment in the ramified morphology [73]. After the occurrence of cerebral ischemia, microglia are activated and transition from the ramified morphology in a resting state to the ameboid morphology in an activated state, initiating the self-propulsive cycle of neuroinflammation and chronic overproduction of inflammatory mediators. This morphological change in microglia is called de-ramification, in which microglial processes are retracted [74]. Therefore, microglial de-ramification after cerebral ischemia is an objective measure of the microglial response. Our study showed that microglial morphologies in the hippocampus of rats were diversified after CCH. The processes of microglia were shortened and reduced, and the cell bodies were rounded, especially in the 2VO and 2VO + medium groups. Next, we quantitatively analyzed the morphology of microglia by AnalyzeSkeleton. We found that the microglia process endpoints/cell were decreased, the microglia process length/cell was shortened, and Iba1 expression was obviously increased in the hippocampus of rats in the 2VO group. These results suggested that after the interruption of cerebral blood flow, microglia were recruited to the hippocampus and activated. EA preconditioning modulated the activation state of microglia, promoting their transition to a resting state and subsequently reducing the inflammatory response. The chemokine CX3CL1 is an endogenous neuronal modulator. CX3CR1, the receptor of CX3CL1, is expressed on the surface of microglia. Activation of microglia leads to increased expression of CX3CL1/CX3CR1, which activates related inflammatory signaling pathways and promotes the release of inflammatory factors such as IL-1β and TNF-α [75–77]. Additionally, CX3CL1/CX3CR1 may also be associated with cognitive dysfunction [78]. In our study, we found that the expression of chemokine CX3CL1/CX3CR1 was increased in the hippocampus of rats in the 2VO group. Compared with that in the 2VO and 2VO + cell groups, the CX3CL1/CX3CR1 expression in the 2VO + cell + EA group was significantly decreased. These results indicated that inflammatory reactions occurred in the hippocampus of 2VO rats. Simple cell transplantation had a certain anti-inflammatory effect, but EA preconditioning combined with cell transplantation had a more significant anti-inflammatory role.

Furthermore, persistent neuroinflammation after CCH can also promote local neuronal apoptosis and aggravate neuronal damage [77]. Therefore, we also detected the expression of apoptotic proteins in the hippocampus. The results indicated that apoptosis was activated in the hippocampus of CCH rats. The up-regulation of Bcl2, the downregulation of Bax, and the increase of Bcl2/Bax ratio in the 2VO + cell + EA group were significant. There is a relationship between Bcl2/Bax and apoptosis. The increase in the Bcl2/Bax indicates that apoptosis is inhibited, and the decrease in the Bcl2/Bax indicates that apoptosis is promoted. The above results manifested that EA preconditioning could inhibit apoptosis and enhance the antiapoptosis effect. Therefore, based on our experimental results of inflammation and apoptosis, we concluded that EA preconditioning showed anti-inflammatory and antiapoptotic effects and ultimately improved the local cerebral ischemic microenvironment.

BDNF is an important member of the neurotrophic factor family, which plays a crucial role in neuroplasticity through the BDNF/TrkB signaling pathway [79]. BDNF and its homologous receptor TrkB have become important upstream regulators of LTP in the hippocampus [80]. Research has indicated that decreased BDNF and TrkB expressions can lead to deficits in learning and memory [81]. BDNF infusion immediately after stroke has been shown to reduce brain tissue damage, improve LTP, and promote functional recovery [82, 83]. In this study, we found that BDNF and TrkB expression in the hippocampus of CCH rats increased in the 2VO + cell and 2VO + cell + EA groups, and this increased trend was more significant in the 2VO + cell + EA group. Thus, the increase of BDNF and TrkB is beneficial to the recovery of cognitive function.

LTP is a typical cellular electrophysiological model used to research the synaptic basis of learning and memory in animals. Generally, used to assess cognitive function, it reflects synaptic plasticity [84, 85]. The percent increase in the fEPSP slope is usually used as an evaluation index of LTP. The greater the increase in the fEPSP slope, the stronger the learning and memory function. According to a previous study, the LTP value typically increases by 150%–200% of the pre-evoked fEPSP value [85]. In our study, the LTP value measured in the 2VO group was 103.1% ± 2.316%, which was lower than 150%. This finding implied that the LTP of the CA3–CA1 pathway in the 2VO group was inhibited. The result was consistent with previous studies that CCH generally led to impaired LTP [43]. LTP value was 136.2% ± 1.603% in the 2VO + cell group, suggesting that MGE neural progenitor transplantation could improve LTP. LTP value was 170.8% ± 15.82% in the 2VO + cell + EA group, which was higher than 150%. These results demonstrated that EA preconditioning enhanced the efficacy of cell transplantation, reversed the inhibition of LTP, and improved the learning and memory dysfunction of CCH rats. We utilized the Morris water maze task to validate that MGE neural progenitor transplantation could improve learning and memory function in CCH rats, while EA preconditioning enhanced the transplantation efficacy in our previous study [22]. In this study, we found that the effect of EA preconditioning combined with cell transplantation on the expression of BDNF and TrkB was consistent with the improvement trend of LTP. Therefore, we speculated that the improvement of cognitive function in CCH rats may be related to the increased expression of BDNF and TrkB. In subsequent studies, we need to verify the mechanism further through gene knockout or overexpression.

## 5. Conclusion

To summarize, EA preconditioning ameliorates the ischemic microenvironment and enhances the efficacy of MGE neural progenitor transplantation to improve the LTP of CCH rats. Our study provides theoretical support for the future clinical application of EA combined with cell transplantation. However, the limitations of this experiment include that the observation period of the transplanted cells is not long enough, and whether the transplanted cells can survive for a long time and play a stable role is still unknown. We hope to observe the survival rate of transplanted cells, the proportion of neuronal differentiation, and the integration with the host neural circuit in the future, and further explore the mechanisms of EA preconditioning to enhance the efficacy of cell transplantation.

## Data Availability

The data used to support the findings of this study are available from the corresponding author upon reasonable request.

## Nomenclature

ACSF: Artificial cerebrospinal fluid
AD: Alzheimer's disease
BDNF: Brain-derived neurotrophic factor
CCH: Chronic cerebral hypoperfusion
EA: Electroacupuncture
ESCs: Embryonic stem cells
fEPSP: Field excitatory postsynaptic potential
GABA: Gamma-aminobutyric acid
hESCs: Human ESCs
HFS: High-frequency stimulation
iPSCs: Induced pluripotent stem cells
LTP: Long-term potentiation
MEFs: Mouse embryonic fibroblasts
MGE: Medial ganglionic eminence
SD: Sprague–Dawley
SHH: Sonic hedgehog
TrkB: Tyrosine receptor kinase B
2VO: Two-vessel occlusion
VACHT: Vesicular acetylcholine transporter
VCI: Vascular cognitive impairment.

## Supporting Information

Supporting Information Supporting Information mentioned above include three figures. Figure S1 shows the generation of medial ganglionic eminence (MGE) neural progenitors from human embryonic stem cells (hESCs) in vitro. Figure S2 shows the schematic diagrams of Baihui (GV20) and Dazhui (GV14) acupoints. Figure S3 shows the whole experiment flowchart. Figure S4 shows the statistics of the positive rate of NKX2.1 after adherent culture for 2 days.
