# Supplementary material for: Electroacupuncture Preconditioning Ameliorates the Ischemic Microenvironment to Improve Long-Term Potentiation in Chronic Cerebral Hypoperfusion Rats With MGE Neural Progenitor Transplantation
Source: Neural Plast. 2025 Sep 10;2025:9933756. doi: 10.1155/np/9933756 (PMC12443521; doi:10.1155/np/9933756)
Supplement: Supporting Information — mentioned above include three figures. Figure S1 shows the generation of medial ganglionic eminence (MGE) neural progenitors from human embryonic stem cells (hESCs) in vitro. Figure S2 shows the schematic diagrams of Baihui (GV20) and Dazhui (GV14) acupoints. Figure S3 shows the whole experiment flowchart. Figure S4 shows the statistics of the positive rate of NKX2.1 after adherent culture for 2 days. [file 9933756.f1.docx]

**SUPPLEMENTARY FIGURES**

**
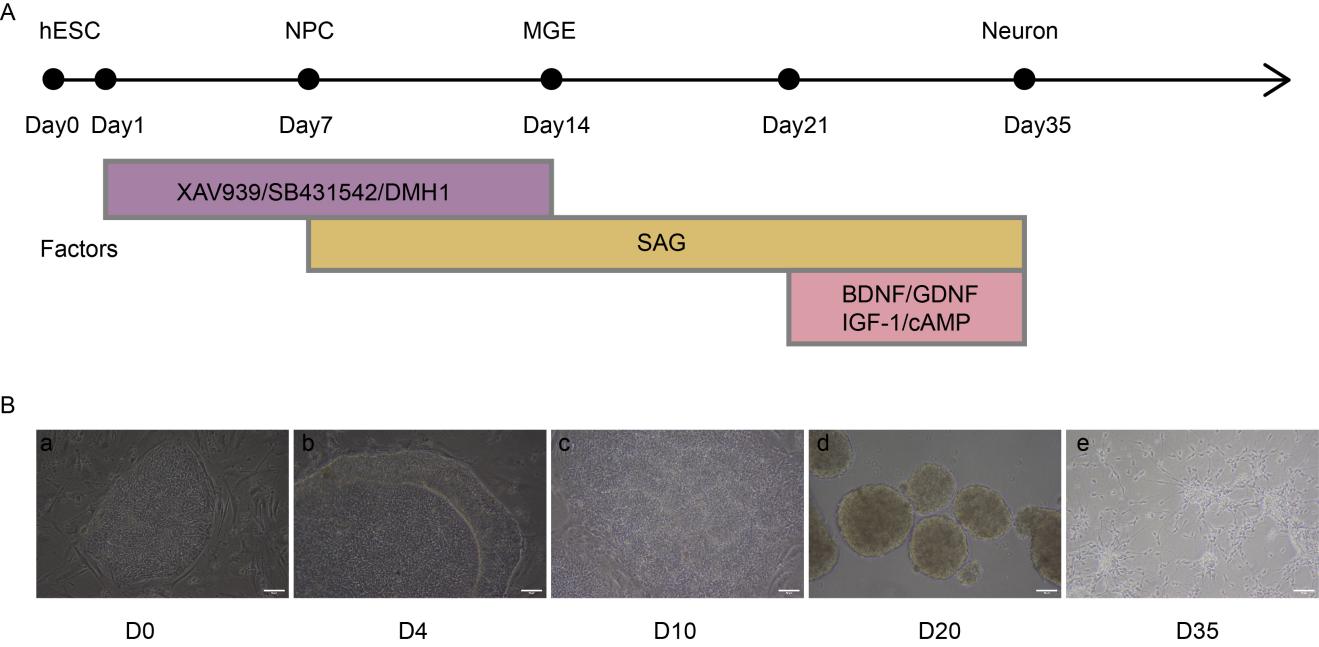
**

**Figure. S1** Generation of medial ganglionic eminence (MGE) neural progenitors from human embryonic stem cells (hESCs) in vitro. (A) Flow chart of hESCs differentiation by dual SMAD inhibition protocol. (B) Morphologies of cells at different stages of differentiation under light microscope. (Bar=50μm)


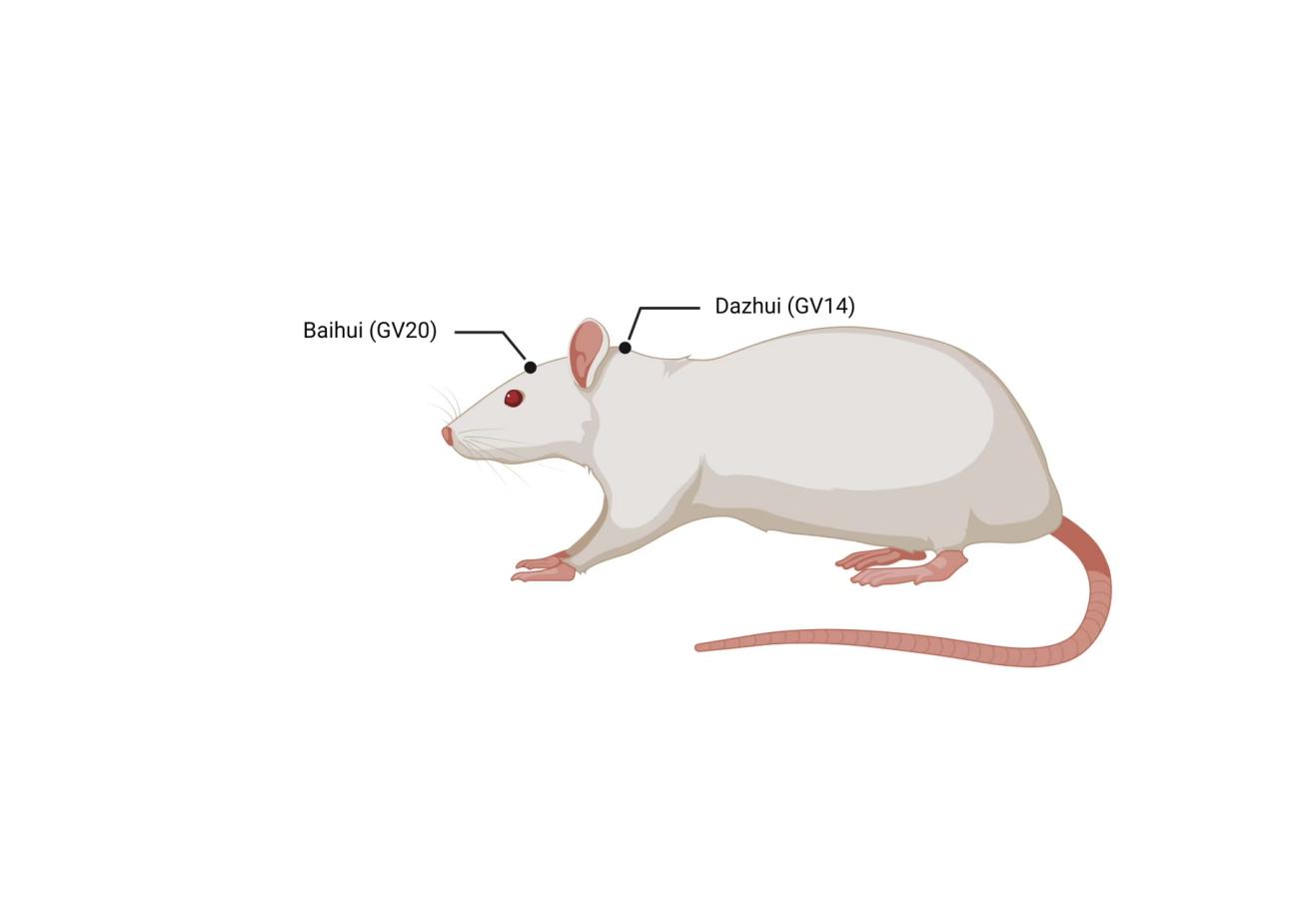


**Figure. S2**. Schematic diagrams of Baihui (GV20) and Dazhui (GV14) acupoints


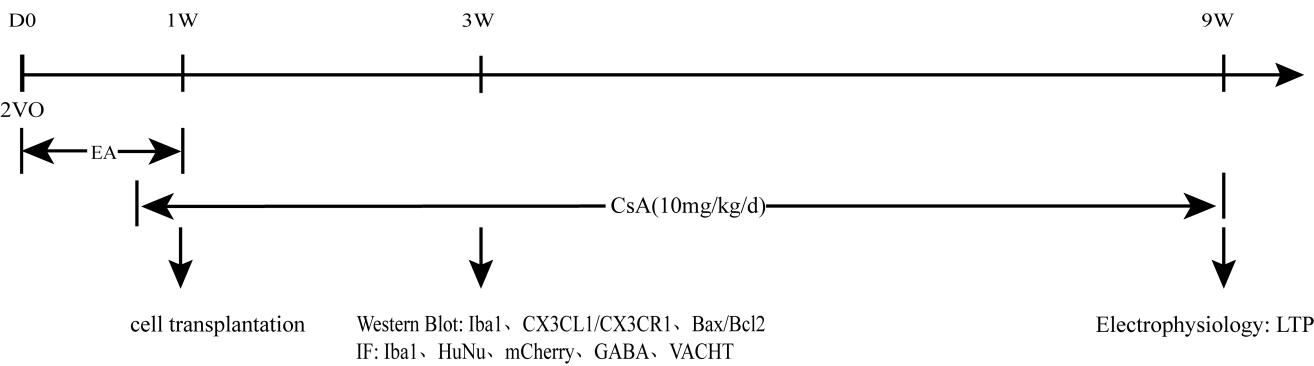


**Figure. S3** The whole experiment flowchart.


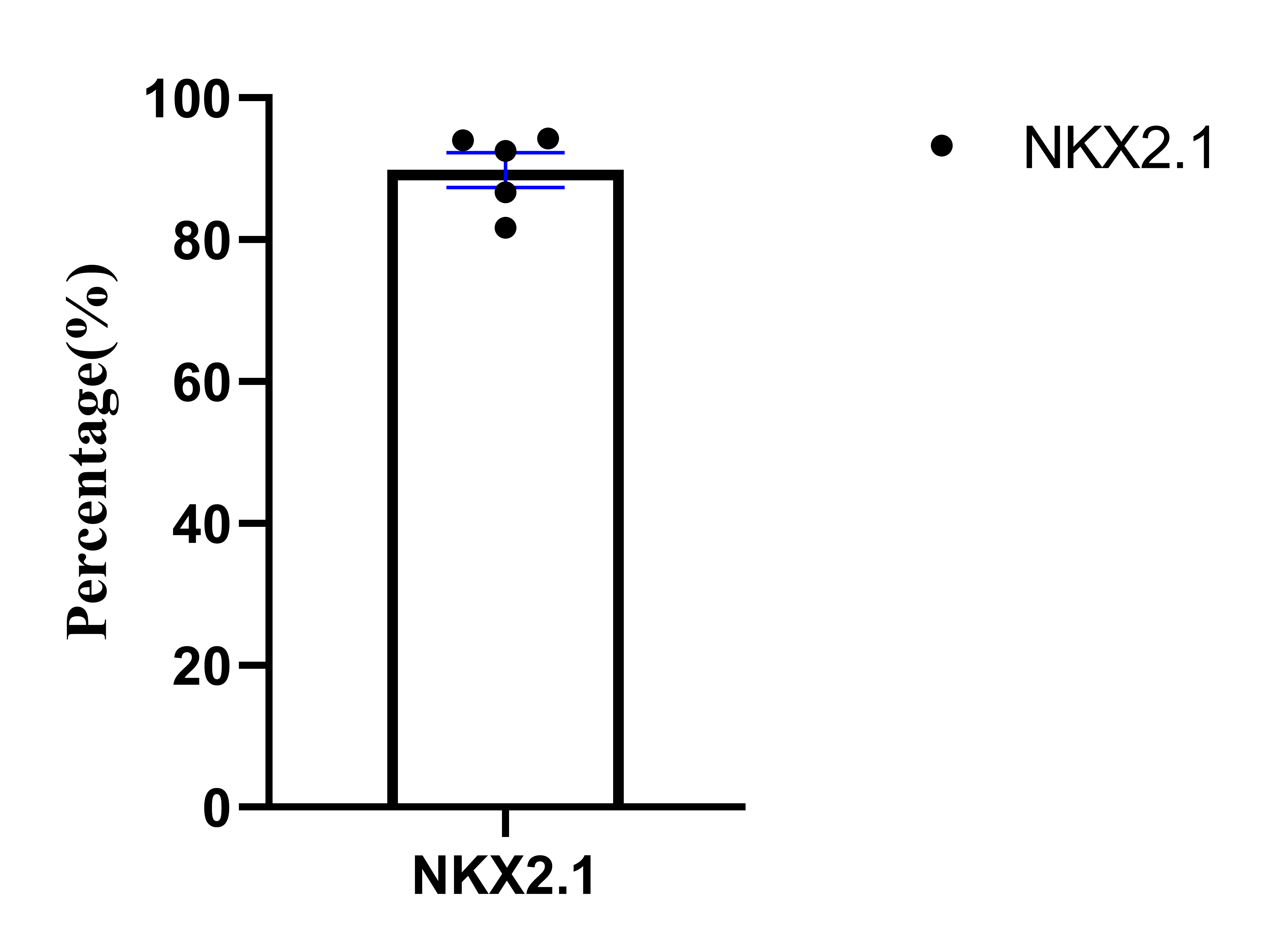


**Figure. S4** Statistics of the positive rate of NKX2.1 after adherent culture for 2 days.
